# Inhibition of *PTTG1* suppresses proliferation and promotes differentiation of neuroblastoma cells by inducing autophagy

**DOI:** 10.1007/s00383-025-06246-w

**Published:** 2025-11-20

**Authors:** Lihua Yuan, Xiaobo Wang, Kanglin Dai, Kenneth Kak Yuen Wong

**Affiliations:** 1 Division of Pediatric Surgery, Department of Surgery, The University of Hong Kong Shenzhen Hospital, Shenzhen, China; 2 Division of Pediatric Surgery, Department of Surgery, The University of Hong Kong, Queen Mary Hospital, Pokfulam Road, Hong Kong, Hong Kong SAR, China; 3 Department of Hematology, The Seventh Affiliated Hospital, Sun Yat-sen University, Shenzhen, China

**Keywords:** Neuroblastoma, PTTG1, Autophagy, Proliferation, Differentiation

## Abstract

**Purpose:**

*PTTG1* is an oncogene that is highly expressed in various cancers and is involved in regulating the cell cycle in neuroblastoma (NB) cells. However, the specific role of *PTTG1* in NB has not been extensively reported. We undertook this study to investigate the expression of *PTTG1* in various NB cell lines to identify the gene expression patterns.

**Methods:**

Small interfering RNA (siRNA) targeting *PTTG1* was designed and used to transfect NB cells. Cell proliferation levels, wound healing and transwell experiments were undertaken to assess the invasion and migration abilities of transfected and control NB cells. Western blot, PCR, and immunofluorescence experiments were utilized to detect the expression of migration-related proteins, differentiation-related proteins, and autophagy-related proteins in NB cells. Different doses of the autophagy inhibitor 3-methyladenine (3-MA) were used for validating the mechanism.

**Results:**

High expression of *PTTG1* was seen in three types of NB cell lines, with the most significant levels observed in SK-N-SH cells. Interference of *PTTG1* significantly inhibited the activity of SK-N-SH cells, reducing their proliferation, invasion, and migration abilities, and was accompanied by a decrease in MMP2 and MMP9 protein expression. In addition, there was enhancement of fluorescence intensity of the differentiation marker TUBB3 and the autophagy marker LC3II, and upregulated the protein expression and mRNA levels of GAP43, TH, MEG, TUBB3, LC3II/LC3I, and beclin1, while downregulated the expression levels of P62 and mTOR. After applying the autophagy inhibitor 3-MA, the regulation of SK-N-SH cell proliferation and differentiation by *PTTG1* interference was significantly reduced.

**Conclusions:**

*PTTG1* is highly expressed in various NB cells. Interfering with *PTTG1* induces autophagy, thereby inhibiting SK-N-SH cell proliferation and promoting differentiation.

## Introduction

Neuroblastoma (NB) is a malignant tumor of the sympathetic nervous system that originates from neural crest cells. It is the most common extracranial solid tumor in children, accounting for about 15% of childhood cancer deaths [1, 2]. The clinical manifestations of NB vary depending on the location of the primary tumor. Typically, patients present with discomfort, fever, weight loss, and bone pain at the time of diagnosis [3]. Currently, treatment methods for NB mainly include surgical resection, chemotherapy, radiation therapy, high-dose chemotherapy with stem cell transplantation, retinoic acid therapy, and immunotherapy [4, 5]. Statistics show that the survival rate for low- and intermediate-risk NB patients is over 95%, whereas the 5-year survival rate for high-risk NB patients is less than 50% [6, 7]. Therefore, a comprehensive understanding of the molecular biology mechanisms underlying the proliferation and differentiation of NB is necessary to further improve patient prognosis.

Pituitary tumor-transforming gene 1 (*PTTG1*) is considered an oncogene, highly expressed in various cancers such as bladder cancer, ovarian cancer, and breast cancer [8–10]. *PTTG1* promotes tumor cell proliferation, invasiveness, epithelial-mesenchymal transition, and angiogenesis [11, 12]. Current research indicates that *PTTG1* is involved in cell differentiation and transformation. It is concurrently expressed in fetal liver and placenta, as well as in pituitary adenomas, breast cancer, and NB [13]. PCNA-associated factor (PCLAF) promotes the G1/S cell cycle progression of NB cells through *PTTG1* [14], thereby suggesting the involvement of *PTTG1* in the NB process. However, detailed reports on how *PTTG1* affects the proliferation and growth of NB have not been published.

Autophagy, as a common mechanism for maintaining cellular homeostasis in eukaryotic cells, plays a dual role in either inhibiting or promoting cancer progression [15]. Studies have shown that Matrine can inhibit the proliferation of NB cells by activating autophagy [16], while TP53BP2 can suppress NB cell proliferation by inducing autophagy [17]. Norcantharidin inhibits the growth of SK-N-SH NB cells by inducing autophagy [18]. This indicates that autophagy is involved in regulating the proliferation and growth of NB cells. Furthermore, research has shown that *PTTG1* knockout can inhibit the activation of the mTOR signaling pathway through autophagy regulation [12, 19], suggesting a potential relationship between PTTG1 and autophagy regulation in tumor cells.

Therefore, considering the crucial oncogenic role of *PTTG1* in tumor progression, this study will further investigate the role of *PTTG1* in NB, aiming to explore whether *PTTG1* inhibits NB cell proliferation and growth by regulating autophagy.

## Materials and methods

### Cell culture

Human embryonic kidney 293 (HEK293) cells, SK-N-BE, IMR-32 and SK-N-SH NB cell lines were provided by the Type Culture Collection of the Chinese Academy of Sciences (Shanghai, China). HEK293 cells were used as a control. The cells were cultured in RPMI-1640 medium (Thermo Fisher scientific, USA) containing 10% fetal bovine serum (FBS) and 1% penicillin/streptomycin at 37 °C in an incubator with 5% CO_2_.

### Cell transfection

The small interfering RNA of *PTTG1* (SiRNA-PTTG1-1/2) and its negative control (SiRNA-NC) were provided by GenePharma Biotech (Shanghai, China). The cells were inoculated in a 6-well plate at a density of 1 × 10^5^ cells per well. Transfection was performed using the Lipofectamine 2000 according to the manufacturer’s instructions (Invitrogen) when the cells grew to 70–80% confluence. The cells were cultured at 37 ˚C with 5% CO_2_ for 4 h and then the medium was changed to continue culture for 48 h. Western blot (WB) was adopted to detect the transfection efficiency. The 5mM and 10mM [20, 21] autophagy inhibitor 3-methyladenine (3-MA, HY-19312, MedChemExpress, USA) was dissolved into the culture medium for autophagy mechanism verification.

### CCK-8 assay

Cell proliferation assays were carried out using a cell counting kit-8 (CCK-8). Transfected cells in 96-well plates and adjusted the cell density to 6 × 10^3^ cells/well. Each well was cultivated for extra 2 h at 37˚C following addition of 10µL CCK-8 solution (Boster, Wuhan, China). Cell activity was assessed by estimating OD450 nm value using a microplate reader.

### EDU staining

5-ethynyl-2-deoxyuridine (EdU) staining was used to assess the propagation of NB cells. In brief, transfected NB cells (3 × 10^5^ cells/well) were seeded in a 24-well plate, 10 mM of EdU was added to the medium and cells were incubated for 4 h. Then, the cells were fixed with 4% paraformaldehyde and stained with Hoechst 33,342 for 10 min at room temperature (RT). Images were prepared for observation under a fluorescence microscope (Motic, China) and results were quantified using ImageJ software.

### Cell differentiation assay

After 48 h of transfection, cells were fixed and incubated with the neuronal differentiation marker beta-III tubulin (TUBB3) antibody (1:200, ab18207, abcam). Then, the cells were incubated with anti-rabbit immunoglobulin G conjugated to fluorescein isothiocyanate and 4, 6-diamidino-2-phenylindole. Cell images were captured under a fluorescence microscope. Cell differentiation was assessed by analyzing neurite outgrowth and expressed as the number of neurites per 100 cells [22].

### Wound healing assay

Transfected cells were cultured in 6-well cell culture plates. After the cells were attached, a gap was made using a tip. The gap was observed at 100× magnification and photographs were taken using a microscope (Olympus, Japan) at 0 h, 24 h, and 48 h. The migratory capacity of the cells was assessed by measuring the change in the size of the injured area.

### Transwell assay

Transfected cells were harvested, and 1 × 10^4^ cells in 200µL of DMEM were placed into the upper chamber of Transwell filter membranes precoated with Matrigel. The lower chamber was placed in medium containing 600µL of DMEM with 10% FBS. After 24 h of incubation at 37 °C in a cell culture incubator containing 5% CO2, the cells in the upper chamber were removed. The cells were fixed in methanol for 15 min, stained with crystal violet for 20 min, photographed in five random fields of view under a microscope.

### Reverse transcription-quantitative polymerase chain reaction (RT-qPCR) assay

Total RNA of NB cells was extracted by Trizol (Invitrogen, USA). Reverse transcription was applied by the RevertAid RT Reverse Transcription Kit (MR101-02, Vazyme, USA). RT-PCR was conducted with SYBR Premix Ex Taq II reagent by 7500 RT-PCR System (Applied Biosystems, Foster City, CA, USA). The primers for RT-PCR were obtained from PrimerBank. Gene expression was normalized by GAPDH and relative mRNA expression levels were determined by the 2^−ΔΔCT^ method. The sequences of the primers in this study were presented in Table 1.

**Table 1:** Primer sequences

Gene	Primer	Sequences (5′-3′)
GAPDH	Forward	GATTCCACCCATGGCAAATTC
	Reverse	GTCATGAGTCCTTCCACGATAC
PTTG1	Forward	CCCTCAAACAAAAACAGCCAAG
	Reverse	GGCATCATCTGAGGCAGGAAC
GAP43	Forward	GGCCGCAACCAAAATTCAGG
	Reverse	CGGCAGTAGTGGTGCCTTC
TH	Forward	GGGCTGTGTAAGCAGAACG
	Reverse	AAGGCCCGAATCTCAGGCT
MEG3	Forward	CCTCACCTCCAATTTCCTCTTC
	Reverse	TCCAGCAGCTAACCTCATTAAC
TUBB3	Forward	GGCCAAGGGTCACTACACG
	Reverse	GCAGTCGCAGTTTTCACACTC

### Western blot assay

Cells were collected and lysed with RIPA buffer at 4 °C for 30 min and centrifuged at 12,000 × g for 30 min. The supernatant was collected and quantified by the Protein BCA Quantification Kit (Bio-Rad Laboratories, USA). 20 µg protein was loaded into the well and separated with sodium dodecyl sulfate polyacrylamide gel electrophoresis (SDS-PAGE), then the gel was transferred to the PVDF membrane (Amersham Biosciences). The blots were blocked with 5% BSA at RT for 60 min and then blots were incubated with primary antibody at 4 °C overnight. The primary antibody including anti-PTTG1 (AF0354, 1:1000, Affinity), anti-LC3 (AF5402, 1:1000, Affinity), anti-mTOR (DF6308, 1:1000, Affinity), anti-p62/SQSTM1 (P62) (DF6657, 1:1000, Affinity), anti-GAP43 (DF14676, 1:1000, Affinity), anti-tyrosine hydroxylase (TH) (AF6113, 1:1000, Affinity), anti-MEG3 (1:1500, Affinity, DF7438), anti-TUBB3 (ab18207, 1:1500, Abcam) and anti-GAPDH (ab9485, 1:3000, Abcam). Then the membranes were washed in TBST and incubated with horseradish peroxidase (HRP)-conjugated secondary antibodies for 1 h at RT. The protein signals were exposed using ECL reagent (Beyotime) and the immunoblotting signals were quantitatively analyzed by ImageJ software.

### Immunofluorescence

Cells were seeded in 24-well plates (8 × 10^4^ cells/well) and fixed with 4% paraformaldehyde (Sigma-Aldrich). Then, the cells were incubated with 5% Tween-20 for 2 h and blocked with 10% normal goat serum for 1 h. Then diluted primary antibody anti-LC3II (1:100, Abcam) was added and incubated overnight at 4℃. Followed by incubation with a secondary antibody (1:100, DAR-546, Abcam) for 2 h. After washing with PBS, the cell nuclei were re-stained with DAPI solution for 30 min, and observed under a fluorescence microscope (Nikon, Austria).

### Statistical analysis

Data were analyzed using SPSS 22.0 software and statistical comparisons of multiple variables were made by one-way analysis of variance (ANOVA) and the Tukey test. All the data were expressed as mean ± standard deviation (SD) of three independent experiments. *P* < 0.05 was considered to be statistically significant differences.

## Results

### *PTTG1* interference inhibits proliferation, invasion and migration of SK-N-SH cells

**Fig. 1:**
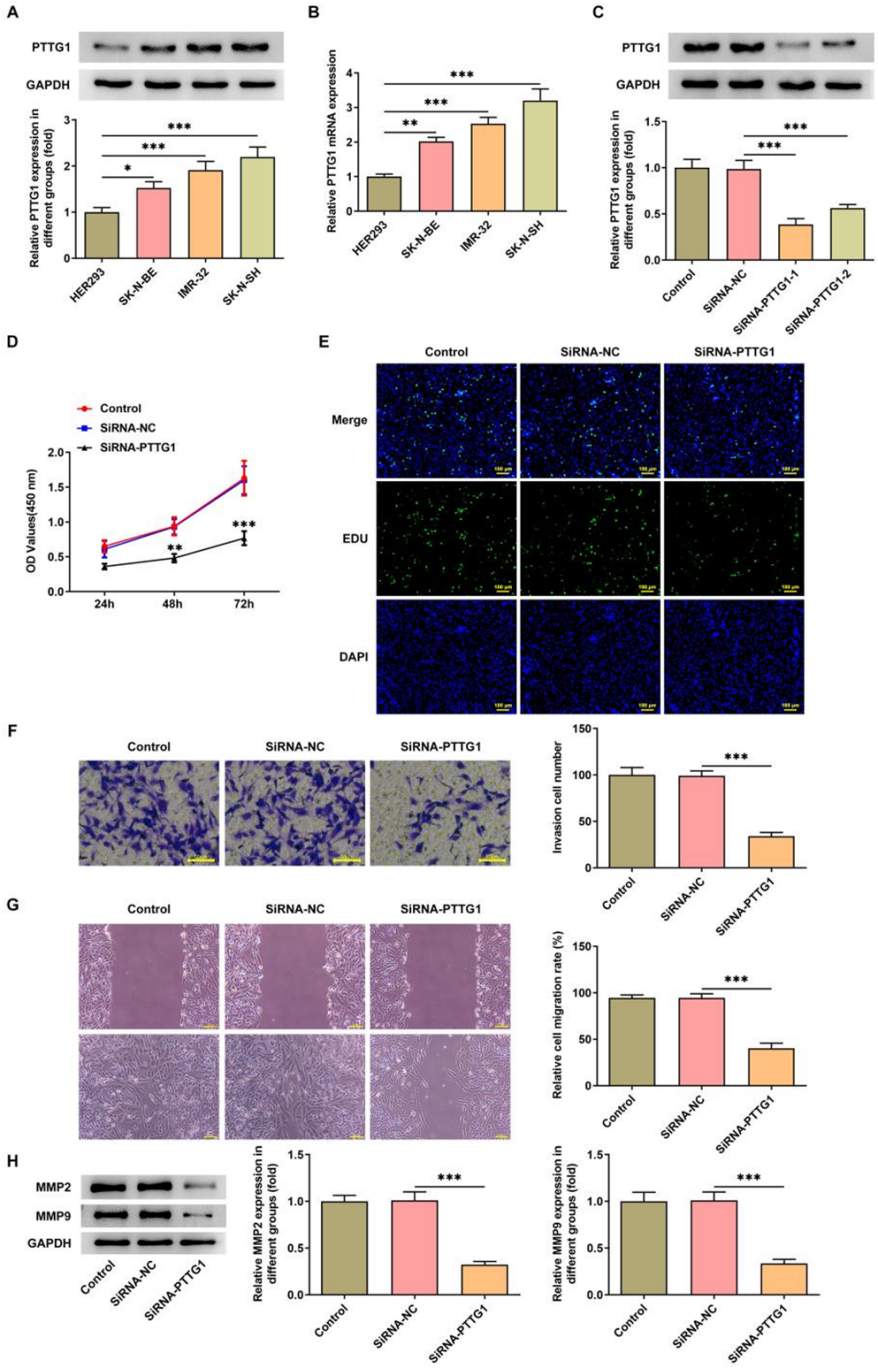
*PTTG1* interference inhibits proliferation, invasion and migration of SK-N-SH cells. PTTG1 protein expression in different NB cell lines (**A**); *PTTG1* mRNA levels in different NB cell lines (**B**); SiRNA transfection efficiency assay (**C**); SK-N-SH cell viability (**D**), proliferative (**E**), invasive (**F**) and migratory (**G**) assays. MMP2 and MMP9 protein expression in SK-N-SH cells (**H**)

We investigated the expression pattern of *PTTG1* in NB by examining its expression across different NB cell lines. The findings showed a significant increase in both protein expression (Fig. 1A) and mRNA levels (Fig. 1B) in all three NB cell lines when compared to HEK293, with the most notable elevation was noted in the SK-N-SH cell line. siRNA transfection assays for PTTG1 showed that SiRNA-PTTG1-1 had superior interference efficacy (Fig. 1C), thus it was selected for further studies. *PTTG1* interference markedly reduced the cellular viability (Fig. 1D), proliferation (Fig. 1E), invasion (Fig. 1F), and migratory capacity (Fig. 1G) of SK-N-SH cells. Western Blot demonstrated a significant decrease in MMP2 and MMP9 expression in SK-N-SH cells after PTTG1 interference (Fig. 1H).

### *PTTG1* interference promotes SK-N-SH cell differentiation

**Fig. 2:**
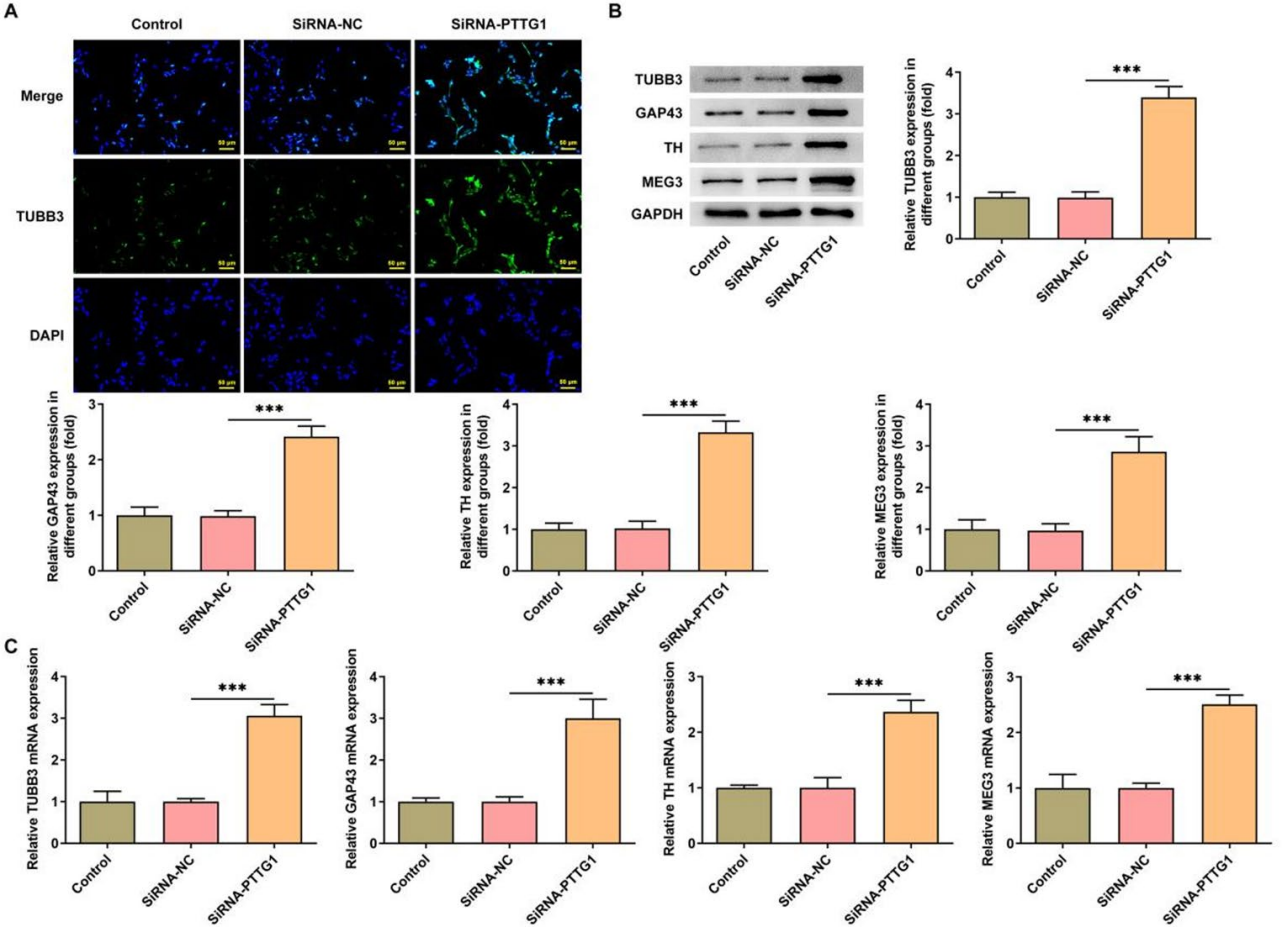
*PTTG1* interference promotes SK-N-SH cell differentiation. TUBB3 fluorescence intensity (**A**); protein expression (**B**) and mRNA levels (**C**) of tyrosine hydroxylase (TH), maternally expressed gene 3 (MEG3) and b-III microtubulin (TUBB3) in SK-N-SH cells

Beta-III tubulin (TUBB3) serves as a marker for neuronal differentiation. *PTTG1* interference notably increased the fluorescence intensity of TUBB3 (Fig. 2A), indicating a reduced differentiation capacity in SK-N-SH cells. Subsequently, the study investigated the expression of additional differentiation markers. The findings demonstrated that interference of *PTTG1* significantly increased the protein expression (Fig. 2B) and mRNA levels (Fig. 2C) of growth-associated protein 43 (GAP43), tyrosine hydroxylase (TH), maternally expressed gene 3 (MEG3), and TUBB3.

### *PTTG1* interference promotes autophagy in SK-N-SH cells

**Fig. 3:**
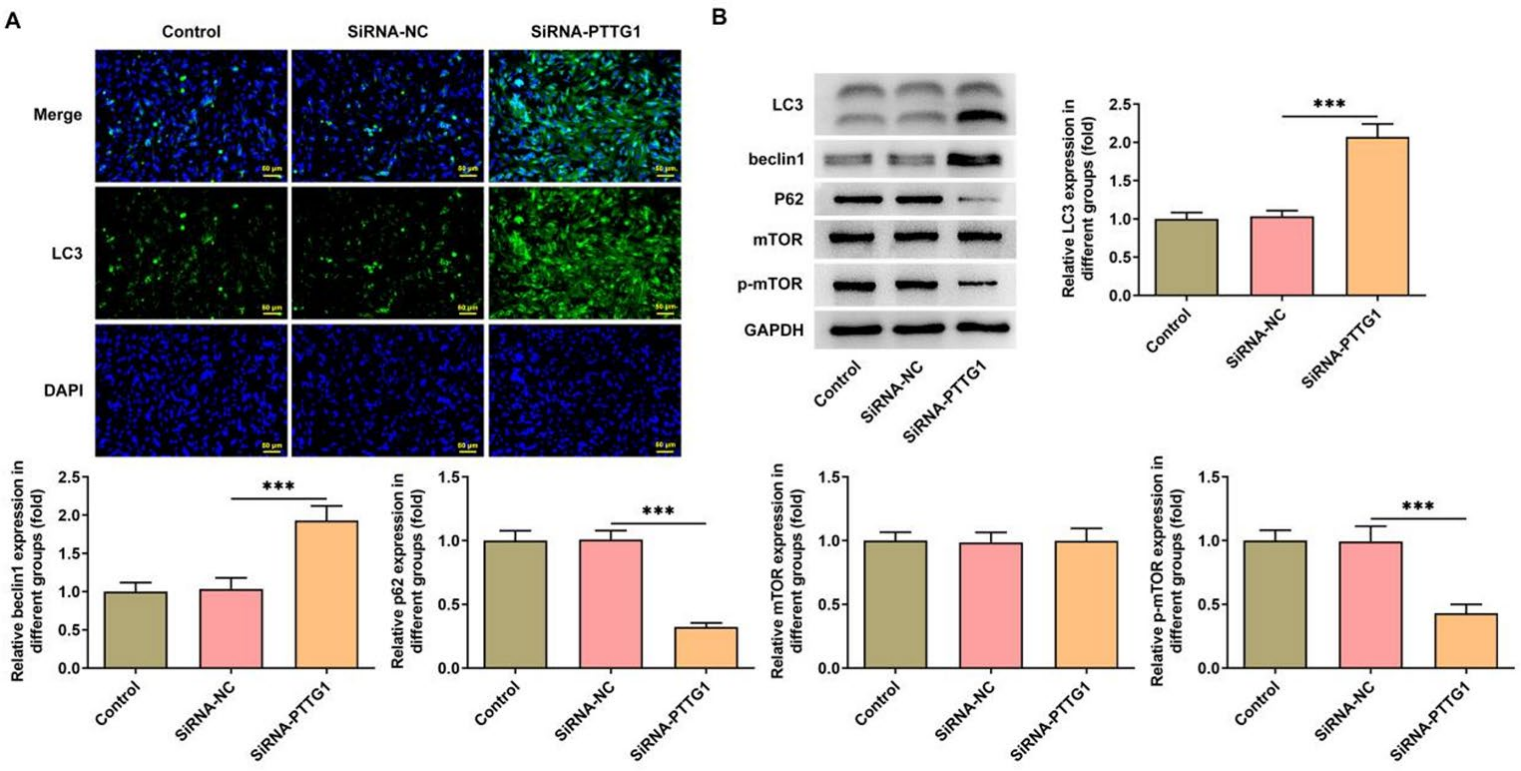
*PTTG1* interference promotes autophagy in SK-N-SH cells. Immunofluorescence detection of LC3II expression (**A**); autophagy-related proteins LC3II/LC3I, Beclin1, P62, and mTOR expression (**B**)

The level of autophagy in SK-N-SH cells was assessed by examining the expression of autophagy-related proteins, as illustrated in Fig. 3. After *PTTG1* interference, the fluorescence intensity of LC3II increased in SK-N-SH cells (Fig. 3A). WB results showed that the expressions of LC3II/LC3I and beclin1 proteins were increased, while the expression of P62 and mTOR proteins were reduced, when compared to the control and SiRNA-NC groups (Fig. 3B).

### *PTTG1* interference acts through autophagy induction and promotes cell differentiation

**Fig. 4:**
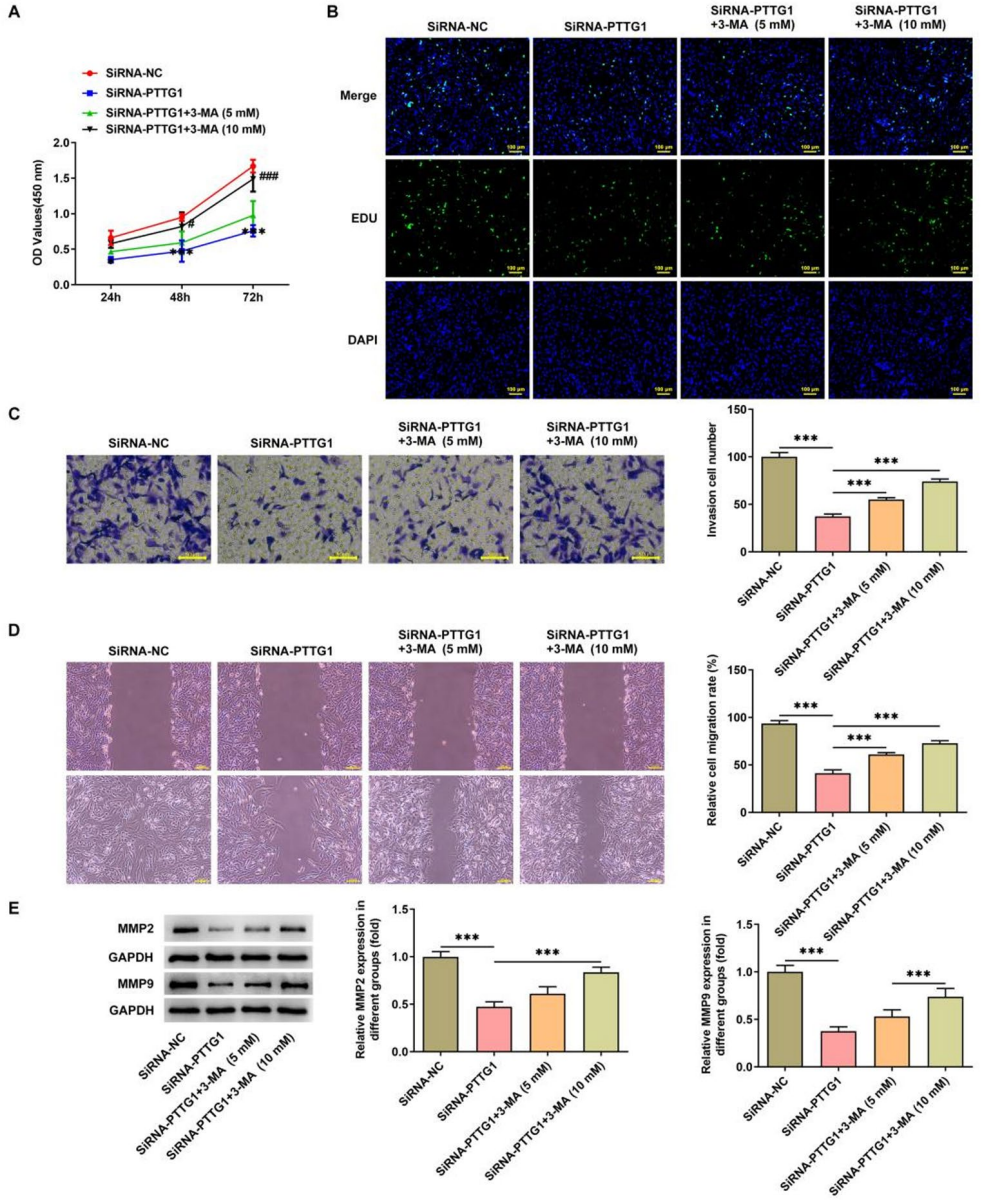
*PTTG1* interference inhibits proliferation, invasion and migration of SK-N-SH cells by inducing autophagy. SK-N-SH cell activity (**A**), proliferation level (**B**), invasive ability (**C**), and migration level (**D**) were evaluated, and MMP2 and MMP9 protein expression was detected in SK-N-SH cells (**E**)

Autophagy inhibitor 3-MA was used to investigate the mechanistic actions of *PTTG1* in SK-N-SH cells. The results indicated that 3-MA mitigated the effects of *PTTG1* interference in SK-N-SH cells. As the dosage of 3-MA increased, the cellular activity (Fig. 4A), proliferation level (Fig. 4B), invasive capability (Fig. 4C), and migratory potential (Fig. 4D) of SK-N-SH cells gradually heightened. Moreover, the expression of MMP2 and MMP9 within the cells showed continuous augmentation (Fig. 4E).

**Fig. 5:**
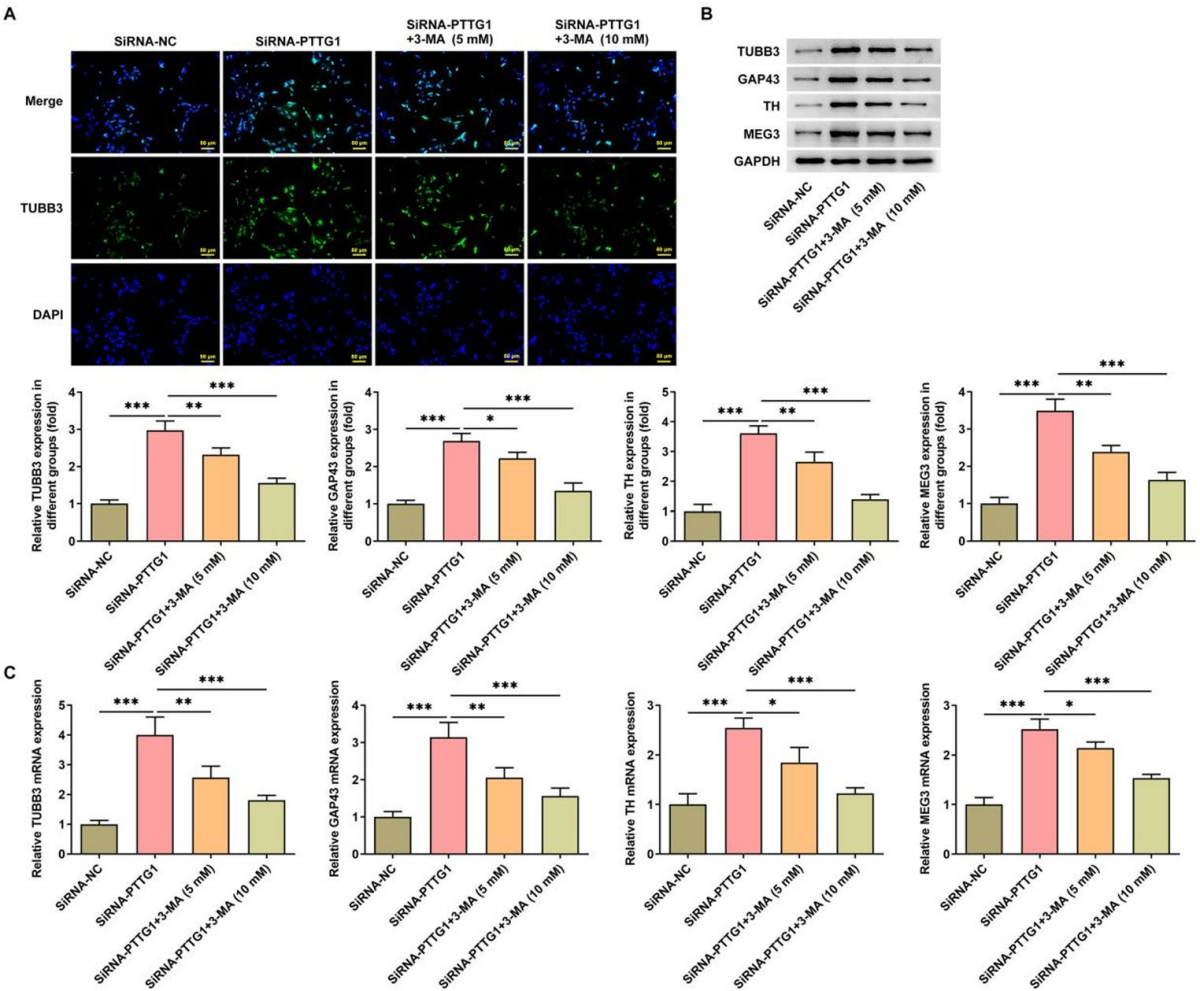
*PTTG1* interference promotes differentiation of SK-N-SH cells by inducing autophagy. TUBB3 fluorescence intensity in SK-N-SH cells (**A**); protein expression (**B**) and mRNA levels (**C**) of the differentiation markers GAP43, TH, MEG3) and TUBB3

Finally, we investigated the effect of *PTTG1* disruption on the differentiation of SK-N-SH cells after applying an autophagy inhibitor. The results showed that treatment with 5mM or 10mM of 3-MA led to a decrease in the fluorescence intensity of TUBB3 in cells (Fig. 5A). Protein expression levels of differentiation markers GAP43, TH, MEG, and TUBB3 (Fig. 5B), as well as mRNA levels (Fig. 5C), significantly decreased.

## Discussion

NB is the most common extracranial tumor in children, and identifying new tumor targets offers the potential to introduce innovative therapeutic strategies aimed at improving the survival and cure rates of high-risk NB patients [23]. This study has revealed increased expression of *PTTG1* in various NB cells. Interference of *PTTG1* significantly reduced the vitality of SK-N-SH cells, while also reducing cellular proliferation, invasion, and migration, while promoting cellular differentiation and levels of autophagy. The autophagy inhibitor 3-MA mitigated the effects of *PTTG1* interference on SK-N-SH cells.

*PTTG1* has been implicated in cancer invasion and metastasis [24], with documented overexpression across various malignancies. Its role in the migration, invasion, and metastasis of bladder, cervical, and colorectal cancers has been established [9, 25–27]. This study conducted a preliminary exploration of *PTTG1*’s role in NB cells, revealing its high expression similar to that of an oncogene in most cancer cells, and correlating with NB cell proliferation, invasion, and migration. NB exhibits heterogeneity, which correlates with tumor location and differentiation status [28]. A critical event in NB pathogenesis is the differentiation arrest at various stages of neuronal cell development, with inducing NB cell differentiation being one clinical approach to treating NB [29]. Differentiation therapy, which induces NB cell redifferentiation while sparing normal cells and tissues, holds promise for maintenance therapy in high-risk NB patients [30]. In this study, we observed that interfering *PTTG1* upregulated the protein expression of differentiation markers GAP43, TH, MEG, and TUBB3 in SK-N-SH cells. Currently, research on NB primarily focuses on inhibiting tumor cell proliferation, migration, invasion, and inducing apoptosis [31]. This study demonstrates that *PTTG1* regulates not only the proliferation and migration of SK-N-SH cells but also their differentiation level, offering a novel approach for the development of targeted differentiation therapy for NB.

Numerous studies have elucidated the complex interplay between autophagy and metastasis in NB, emphasizing its role in cellular apoptosis, histone modification, angiogenesis, and metabolism [32]. LC3-II is a well-established marker of autophagy, and the ratio of LC3-II/LC3-I serves as a crucial indicator of autophagic activity [33]. Beclin-1, the first mammalian gene implicated in autophagy, along with LC3, plays a central role in the autophagic process [34, 35]. P62, serving as a selective substrate for autophagy, facilitates the formation of protein aggregates for autophagic degradation by interacting with LC3 [36]. mTOR, a critical regulatory pathway for autophagy, has been shown to sustain activation within NB tumors [37]. This study reveals that interfering *PTTG1* significantly increases the protein and mRNA expression of LC3II/LC3I, beclin1, P62, and mTOR, suggesting an elevation in autophagic activity within SK-N-SH cells. Furthermore, treatment with autophagy inhibitors significantly attenuates the regulatory effect of *PTTG1* interference on the proliferation and differentiation of SK-N-SH cells, indicating the involvement of *PTTG1* in NB progression through autophagy mediation.

Nonetheless, this study is preliminary and has certain shortcomings. For example, the choice of the SK-N-SH cell line for investigation was predicated on evaluating *PTTG1* expression across diverse NB cell lines. Furthermore, there are many types of NB cell lines, encompassing MYCN-amplified NB cells and non-MYCN-amplified NB cells, displaying variations in phenotype and differential gene expression [38]. The SK-N-SH cell line falls into the subset of non-MYCN amplification. Further validation is needed to elucidate the role of *PTTG1* in NB cells with MYCN amplification.

The findings of the present study provide preliminary insights and lay the groundwork for future research endeavors. Additionally, the regulation of autophagy involves a myriad of intricate mechanisms. While it has been observed that downregulating *PTTG1* in SK-N-SH cells attenuated mTOR expression, whether the upstream and downstream components of mTOR signaling participate in the actions of *PTTG1* necessitates thorough exploration. Moreover, in vitro experiments cannot fully replicate the complexities of the in vivo environment. To delineate the specific role of *PTTG1* in NB, subsequent in vivo experiments are essential.

In conclusion, this study conducted a preliminary exploration of the role of the oncogene *PTTG1* in NB. The result offers a new perspective for developing novel targets for NB treatment and contributes to a comprehensive understanding of the role of *PTTG1* in tumors.

## Data Availability

The datasets generated for this study are available on request to the corresponding author.
